# Generation of Novel *Plasmodium falciparum* NF135 and NF54 Lines Expressing Fluorescent Reporter Proteins Under the Control of Strong and Constitutive Promoters

**DOI:** 10.3389/fcimb.2020.00270

**Published:** 2020-06-10

**Authors:** Shinya Miyazaki, Annie S. P. Yang, Fiona J. A. Geurten, Catherin Marin-Mogollon, Yukiko Miyazaki, Takashi Imai, Surendra Kumar Kolli, Jai Ramesar, Severine Chevalley-Maurel, Ahmed M. Salman, Geert-Jan A. van Gemert, Youri M. van Waardenburg, Blandine Franke-Fayard, Adrian V. S. Hill, Robert W. Sauerwein, Chris J. Janse, Shahid M. Khan

**Affiliations:** ^1^Department of Parasitology, Leiden University Medical Center, Leiden, Netherlands; ^2^Department of Medical Microbiology, Radboud Center for Infectious Diseases, Radboud University Medical Center, Nijmegen, Netherlands; ^3^Department of Infectious Diseases and Host Defense, Gunma University Graduate School of Medicine, Maebashi, Japan; ^4^Nuffield Department of Medicine, The Jenner Institute, University of Oxford, Oxford, United Kingdom; ^5^TropIQ Health Sciences, Nijmegen, Netherlands

**Keywords:** *Plasmodium falciparum*, malaria, reporter lines, liver stage, NF135, CRISPR/Cas9

## Abstract

Transgenic reporter lines of malaria parasites that express fluorescent or luminescent proteins are valuable tools for drug and vaccine screening assays as well as to interrogate parasite gene function. Different *Plasmodium falciparum* (*Pf* ) reporter lines exist, however nearly all have been created in the African NF54/3D7 laboratory strain. Here we describe the generation of novel reporter lines, using CRISPR/Cas9 gene modification, both in the standard *Pf* NF54 background and in a recently described Cambodian *P. falciparum* NF135.C10 line. Sporozoites of this line show more effective hepatocyte invasion and enhanced liver merozoite development compared to *Pf* NF54. We first generated *Pf* NF54 reporter parasites to analyze two novel promoters for constitutive and high expression of mCherry-luciferase and GFP in blood and mosquito stages. The promoter sequences were selected based on available transcriptome data and are derived from two housekeeping genes, i.e., translation initiation factor SUI1, putative (*sui1*, PF3D7_1243600) and 40S ribosomal protein S30 (*40s*, PF3D7_0219200). We then generated and characterized reporter lines in the *Pf* NF135.C10 line which express GFP driven by the *sui1* and *40s* promoters as well as by the previously used *ef1*α promoter (*GFP@ef1*α, *GFP@sui1, GFP@40s*). The *GFP@40s* reporter line showed strongest GFP expression in liver stages as compared to the other two lines. The strength of reporter expression by the *40s* promoter throughout the complete life cycle, including liver stages, makes transgenic lines expressing reporters by the *40s* promoter valuable novel tools for analyses of *P. falciparum*.

## Introduction

Transgenic rodent and human malaria parasites expressing fluorescent and bioluminescent proteins are used extensively to interrogate parasite biology and malaria pathology as well as tools to evaluate anti-parasite inhibitors and vaccines (Othman et al., 2017). For *Plasmodium falciparum*, transgenic reporter lines have been used to quantify the effect of inhibitors and antibodies on *in vitro* asexual blood stage parasite development (Cui et al., 2008), to evaluate the effect of inhibitors on gametocyte and mosquito stage development (Adjalley et al., 2011; Lucantoni et al., 2013, 2016; Wang et al., 2014; Vos et al., 2015) and to analyze sporozoite infection of hepatocytes in immune-deficient humanized mice engrafted with human liver tissue (Sack et al., 2014; Flannery et al., 2018; Foquet et al., 2018) and sporozoite movement in skin model (Hopp et al., 2019; Winkel et al., 2019).

Most transgenic *P. falciparum* reporter lines have been generated in the widely used laboratory strain NF54 (*Pf* NF54), or its clone 3D7, which originates from an African isolate (Ponnudurai et al., 1981). Reporter expression in the transgenic lines has been driven by various promoters from different genes which are either constitutively expressed in multiple life cycle stages (e.g., the housekeeping genes *ef1*α or *hsp70*) (Talman et al., 2010; Vaughan et al., 2012; Vos et al., 2015) or from stage-specific genes (e.g., *pfs16, gexp02*, or *etramp10.3/peg4*) (Adjalley et al., 2011; Lucantoni et al., 2016; Marin-Mogollon et al., 2019; McLean et al., 2019; Portugaliza et al., 2019). To analyze liver-stage development a parasite line with strong and constitutive expression of the reporter is essential. The *ef1*α promoter drives expression in liver stages and luciferase reporter expression using this promoter has been used to quantify liver infection in humanized mice (Vaughan et al., 2012). However, low activity of the *ef1*α promoter in sporozoites and early liver stages hampers its use for early liver stage analyses (Vaughan et al., 2012; Lucantoni et al., 2016; Marin-Mogollon et al., 2019). Similarly a transgenic *Pf* NF54 reporter line expressing GFP-luciferase under control of the *hsp70* promoter (Vos et al., 2015) has limited reporter expression in liver stages (M.W. Vos. personal communication). Recently, we generated a *Pf* NF54 reporter line (mCherry-Luc@etramp) using the promoter from the *Pfetramp10.3* gene (Marin-Mogollon et al., 2019), which is related to rodent *Plasmodium uis4* gene. In rodent malaria reporter lines the *uis4* promoter has been used to drive high transgene expression in both sporozoites and in liver-stages (Marin-Mogollon et al., 2019). Although expression of the reporter mCherry-luciferase was high in the *Pf* NF54 mCherry-Luc@etramp sporozoites, luciferase expression in liver stages was lower than observed in a *P. falciparum* transgenic line expressing GFP-luciferase under control of the *ef1*α promoter (Marin-Mogollon et al., 2019).

In this study we have examined new *P. falciparum* promoters to drive high reporter expression throughout the parasite life cycle, in particular in mosquito and liver stages. In addition to using the *Pf* NF54 strain we also generated reporter lines in a recently characterized clone from a Cambodian isolate, NF135.C10 (Teirlinck et al., 2013), hereafter referred to as *Pf* NF135. Sporozoites of *Pf* NF135 show a higher invasion rate of hepatocyte *in vitro* and produce more liver merozoites per infected hepatocyte than *Pf* NF54 parasites (McCall et al., 2017). High reporter expression in liver stages of transgenic *Pf* NF135 parasites in combination with increased liver-infectivity should improve liver stage quantification both in inhibitor studies *in vitro* and in humanized mice models, as well as permitting efficient sorting of infected hepatocytes by flow cytometry, as has been achieved using rodent *Plasmodium* reporter lines. To generate the different transgenic *Pf* NF54 and *Pf* NF135 parasites we used a recently developed CRISPR/Cas9 methodology for introducing transgenes into the genome without retaining a drug selectable marker (Mogollon et al., 2016). In this study we describe the characterization of reporter expression in multiple life cycle stages using novel strong promoters of two housekeeping genes (*sui1* and *40s*) and describe for the first time transgenic parasites in the *Pf* NF135 background which demonstrate high GFP expression throughout the parasite life-cycle, including liver-stage development as shown in *in vitro* studies performed in primary human hepatocytes.

## Materials and Methods

### *P. falciparum*: Parasites and *in vitro* Cultivation of Blood Stages

*P. falciparum* NF54 (*Pf* NF54) (Mogollon et al., 2016) and *P. falciparum* NF135 (*Pf* NF135) (Teirlinck et al., 2013) parasites were cultured using standard culture conditions in a semi-automated shaker incubator system as previously described (Mogollon et al., 2016). Fresh human serum and red blood cells (RBC) were obtained from the Dutch National Blood Bank (Sanquin Amsterdam, the Netherlands; permission granted from donors for the use of blood products for malaria research and microbiology; tested for safety). RBC and human serum from different donors were pooled. Cloning of parasites was performed by the method of limiting dilution as described previously (Mogollon et al., 2016). Gametocyte cultures were prepared using standard culture conditions as described (Mogollon et al., 2016). Briefly, parasites from asexual stage cultures were diluted to a final parasitemia of 0.5% and cultures were followed during 14–17 days with daily medium change twice, but without replenishing fresh RBCs. At day 14–17 the cultures were analyzed for stage V gametocytes and fed to *Anopheles stephensi* mosquitoes (see below). The growth rate of asexual blood-stages (parasitemia) of *Pf* NF135 parasite lines was monitored by determination of parasitemia in standard *in vitro* cultures (in a semi-automated shaker incubator system) for a period of 3 days with a starting parasitemia with a parasitemia of 0.1%. Parasitemia was determined by counting infected red blood cells in Giemsa-stained thin blood films in three independent experiments.

### Generation of Transgenic Lines Expressing Reporters

We used the recently developed two plasmid CRISPR/Cas9 method for generation of the transgenic *Pf* NF54 parasites without retaining a selectable marker in the genome (Mogollon et al., 2016). The Cas9 construct pLf0019 (Mogollon et al., 2016), that contains the Cas9 expression cassette and a blasticidin (BSD) drug-selectable marker cassette, was used in combination with the donor DNA plasmids, that are based on the published plasmid pLf0047 (Marin-Mogollon et al., 2019) and contain the *p47* sgRNA/U6 cassette, the *hdhfr-yfcu* drug selectable marker (SM) cassette, the two *p47* homology regions and the transgene expression cassettes. Three different donor DNA constructs were generated, pLf0117, pLf0123, and pLf0128 that contain the mCherry-luciferase (mCherry-Luc) fusion gene under control of the *ef1*α (PF3D7_1357000)*, 40s* (PF3D7_0219200), and *sui1* (PF3D7_1243600) promoters respectively (see below). In addition, two DNA donor constructs were generated, pLf0116 and pLf0127 that contain the GFP gene under control of the *ef1*α and *40s* promoter.

**Figure 1:**
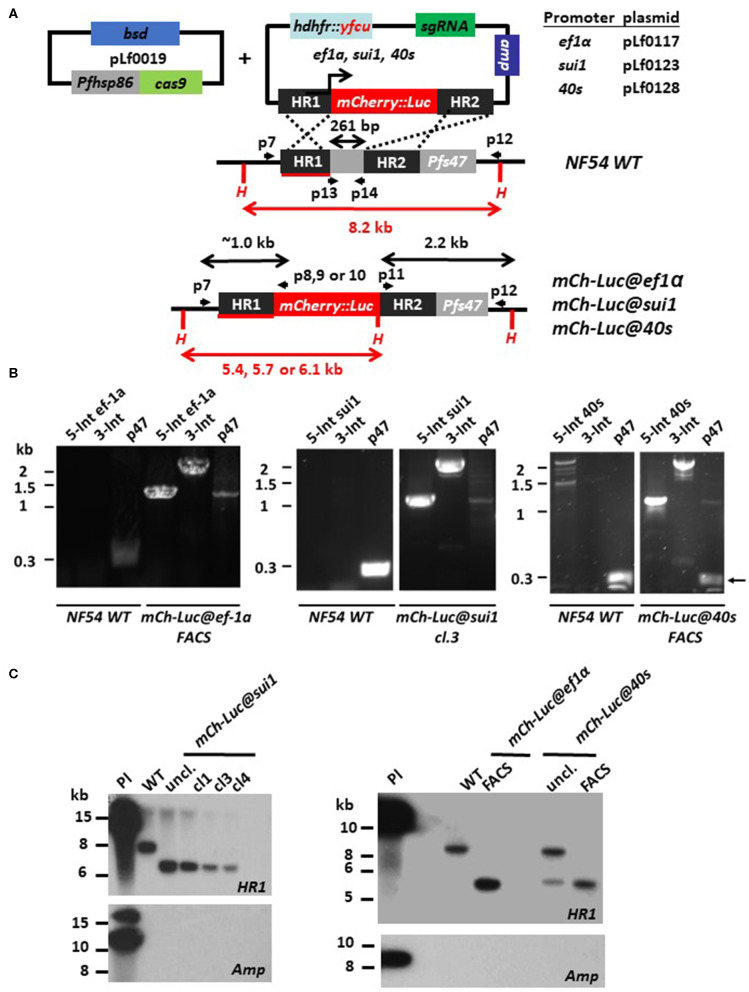
Generation of *P. falciparum* NF54 reporter lines expressing mCherry-luciferase under control of *ef1*α, *sui1*, or *40s* promoter. **(A)** Schematic representation of the Cas9 (pLf0019) and donor DNA plasmids (pL0117, pLf0123, pLf0128) constructs used to introduce the mCherry-luciferase expression cassette into the *Pf* NF54 *p47* gene locus. The mCherry-luciferase fusion gene is under the control of the promoter of the *ef1*α, *sui1*, or *40s* gene. The *p47* homology regions (HR1, HR2) used to introduce the donor DNA (i.e., the mCherry-luciferase expression cassette), location of primers (p), sizes of restriction fragments (H: *Hpa*I; in red), and PCR amplicons (in black) are indicated. Primer sequences (shown in black and bold) are shown in Table S1. WT, wild type; bsd, blasticidin selectable marker (SM); *hdhfr::yfcu*—SM in donor plasmid. **(B)** Diagnostic PCR confirms the correct 5′-integration of the plasmids into the genome of *mCh-Luc@ef1*α*, mCh-Luc@sui1, mCh-Luc@40s* parasites (5-Int; primers p7/p8 for *ef1*α 1,009 bp, p7/p9 for *sui1* 1,106 bp, p7/p10 for *40s* 1,087 bp) and correct 3′-integration (3-Int; primers p11/p12; 2,188 bp). Primer locations and product sizes are shown in **(A)** and primer sequences in Table S1. The arrow indicates PCR product of WT *p47* gene amplified by p13/p14 primers. The weak 1.2 kb band with the p47-primers is a non-specific fragment which is observed in NF54 reporter lines with the mCherry-luc cassette and not in wild type parasites. **(C)** Southern analysis of *Hpa*I restricted DNA to confirm correct integration of the plasmids into *mCh-Luc@sui1* (plasmid pLf0123; left panel) and in *mCh-Luc@ef1*α and *mCh-Luc@40s* parasites (pLf0117 or pLf0128; right panel). Digested DNA was hybridized with a probe targeting the homology region 1 of *p47* [HR1; shown in red; see **(A)**] showing the expected different-sized DNA fragments: WT 8.2 kb; *mCh-luc@sui1* 6.1 kb; *mCh-luc@ef1*α parasites 5.4 kb; *mCh-luc@40s* parasites 5.7 kb (in uncloned parasites the 8,2 kb WT fragment is present). The absence of hybridization of digested DNA with a probe for *ampicillin* (*amp*) confirms the absence of donor DNA plasmid and single crossover integration WT, wild type; uncl., uncloned parasite population; cl, clone; Pl, plasmid.

To generate the transgene-expression cassettes, we first PCR amplified (KOD Hot Start DNA Polymerase, Merck Millipore) 800 bp of the *ef1*α 5′UTR (primers P1/P2, Table S1), 1,493 bp of the *sui* 5′UTR (primers P3/P4, Table S1), and 1,097 bp of the *40s* 5′UTR (primers P5/P6, Table S1) using *Pf* NF54 genomic DNA as template. These 5′UTR regions (promoters) were introduced by SacII/XhoI sites into either the intermediate plasmid, pLf0188 that contains an mCherry-luciferase fusion gene (under control of the *gapdh* promoter; Marin-Mogollon et al., 2019) or in the intermediate plasmid pLf0187 that contains a GFP gene (under control of the *gapdh* promoter; Marin-Mogollon et al., 2019). The transgene expression cassettes from these plasmids were subsequently removed by ApaI/SacII digestion and ligated into plasmid pLf0047, resulting in the final mCherry-Luc donor DNA plasmids pLf0117, pLf0123, and pLf0128 (Figure 1 and Figure S1) and the final GFP donor DNA plasmids pLf0116, pLf0122, and pLf0127 (Figures S1, S2).

Plasmids for transfection were isolated from 250 ml cultures of *Escherichia coli*, XL10-Gold Ultracompetent Cells (Stratagene) by maxi-prep [using HiSpeed1Plasmid Maxi Kit (Qiagen 1)] to generate 50 μg of DNA used per transfection. Transfection with CRISPR/Cas9 constructs was performed using the “spontaneous uptake method” as previously described (Deitsch et al., 2001). Briefly uninfected red blood cells (300 μl of packed RBCs) were transfected with CRISPR constructs (a mixture of ~50 μg of each circular plasmid (Cas9 and donor DNA constructs in 200 μl cytomix) using the Gene Pulser Xcell electroporator (BioRad) with a single pulse (310 V, 950 μF and ∞ Capacity). Subsequently, the transfected RBCs were washed with complete medium and mixed with *Pf* NF54-infected RBCs to a parasitemia of 0.1–0.5% and a hematocrit of 5%. These cells were transferred into a 10 ml culture flask of the semi-automated shaker system.

Selection of transfected *Pf* NF54 parasites was performed by applying double positive selection for a period of 6–19 days by adding the drugs WR99210 (2.6 nM) and BSD (5 μg/ml) starting 3 days after transfection. After the days of treatment with double drugs, parasites were maintained in drug-free medium until parasites were detectable in Giemsa-stained thin blood films (~12–24 days). In independent experiments, we obtained parasite populations *mCh-Luc@ef1*α (Pf-Exp. 156), *mCh-Luc@sui1* (Pf-Exp. 159), *mCh-Luc@40s* (Pf-Exp. 187), *GFP@ef1*α (Pf-Exp. 155), and *GFP@40s* (Pf-Exp. 175) expressing mCherry or GFP as determined by fluorescence microscopy analysis of mixed blood stages. For the *mCh-Luc@sui1* parasites, different clonal lines were obtained by limiting dilution (see below). For *mCh-Luc@ef1*α, *mCh-Luc@40s* line, mCherry-positive cells were collected by flow cytometry (FACS) sorting (see below) using mixed blood stages, resulting in parasite populations in which more than 90% of blood stages were mCherry-positive (determined by fluorescence microscopy).

Selection of transfected *Pf* NF135 parasites was performed by applying double positive selection by adding the drugs pyrimethamine (100 ng/ml) and BSD (5 μg/ml). After transgenic parasites stably grew in the semi-automated shaker system (usually after 5 to 8 days of positive selection), both drugs were removed from the cultures for 2–4 days, and negative selection was applied by addition of 5-Fluorocytosine as described except for *GFP@40s*_135_ (Mogollon et al., 2016). In independent experiments we obtained the following parasite populations *GFP@ef1*α_NF135_ (Pf-Exp. 160), *GFP@40s*_NF135_ (Pf_Exp. 165), and *GFP@sui1*_NF135_ (Pf_Exp. 164) expressing GFP as determined by fluorescence microscopy analysis of mixed blood stages. For the *GFP@40s*_NF135_ and *GFP@sui1*_NF135_ populations we performed two rounds of FACS-sorting (see below) to enrich for transgenic parasites expressing GFP, resulting in parasite populations where more than 99% of the blood stages were GFP positive (determined by fluorescence microscopy). From the *GFP@ef1*α_NF135_ population we obtained a GFP-expressing clone (clone 2) by the method of limiting dilution (see below).

### Genotyping of Transgenic *P. falciparum* Reporter Lines

For genotyping and Southern blotting genomic DNA was isolated as previously described using phenol/chloroform DNA isolation (Mogollon et al., 2016). Correct integration of reporter constructs was analyzed by PCR amplification of the fragments: 5′-integration, 3′-integration and the *p47* gene (see Table S1 for primer sequences). The PCR fragments were amplified using KOD Hot Start Polymerase (Merck Millipore) following standard conditions with annealing temperatures of 50, 55, 60°C for 10 s and an elongation step of 68°C. Southern blot analyses of digested DNA were performed with *Hpa*I-digested genomic DNA (overnight at 37°C). Digested DNA was hybridized with probes targeting the *p47* homology region 1 (HR1), amplified from NF54 genomic DNA by PCR (primers P19/P20) and a second probe targeting ampicillin (Amp) gene, amplified from a plasmid by PCR (primers P21/P22; see Table S1 for primer sequences).

### Flow (FACS) Sorting of GFP- and mCherry-Expressing Parasites

To enrich for transfected parasites obtained after drug selection (see above) we selected GFP- and mCherry-expressing blood stages by FACS sorting. Briefly, 200 μl samples of mixed blood stage cultures (parasitemia 1 to 8%) were diluted in 1 ml of complete medium supplemented with 3% of human serum and 1% penicillin/streptomycin (Gibco). Parasites were sorted using a BD FACSAria™ III (Becton Dickinson, Mountain View, CA, USA). RBCs were selected by gating on Forward and Side Scatter parameters (FSC and SSC, respectively). Doublets were excluded by using FSC-Area and FSC-height parameters. For GFP, a blue laser (488 nm excitation) was used in combination with band pass filter 530/30 nm. For mCherry, a yellow/green laser (561 nm excitation) was used in combination with band pass filter 615/20 nm. A total of 1–3 × 10^3^ fluorescent cells were selected and collected in a well of a 24 wells plate in complete medium supplemented with 20% of human serum and 1% penicillin/streptomycin. Then the sorted samples were diluted to 10 cells or 100 cells per culture and transferred to 10 ml flask. FACS-sorted parasites were cultured under standardized conditions for a period of 7 to 14 days in the semi-automated shaker system. At a parasitemia of 5 to 10%, parasites were collected for genotyping, fluorescence analyses (see below) and storage in liquid nitrogen.

### Flow Cytometric Analysis of GFP-Expression of Asexual Blood Stages

The relative GFP-fluorescence intensities of different asexual blood stages were determined as previously described (Janse and van Vianen, 1994; Mogollon et al., 2016) with a few modifications. Briefly, triplicate samples of 100 μl of infected RBC were collected from cultures that had been synchronized with sorbitol as described previously (Mogollon et al., 2016). Samples were collected at 12 and 42 h after synchronization and resuspended in 3 ml of culture medium containing 3% serum. Cells were stained with the DNA-specific dye Hoechst-33258 at 37°C for 30 min (Janse and van Vianen, 1994; Mogollon et al., 2016). GFP- and Hoechst-fluorescence intensity was determined using a LSRII flow cytometer (Becton Dickinson, Mountain View, CA, USA), data were generated using the FACS DIVA software (Becton Dickinson) and analyzed with FlowJo software (Treestar, Ashland, OR, USA). Per sample 100,000 cells were evaluated and RBCs were selected by gating on FSC and SSC and excluding doublets. Excitation of cells for Hoechst-33258 was performed with a UV laser (355 nm) and band pass filter 450/50 nm and for GFP with a blue laser (488 nm) and a band pass filter of 530/30 nm. The GFP fluorescence intensity was determined of the haploid blood stages [rings and trophozoites; Gate 1 (G1)] and polyploid blood stages [schizonts; Gate 2 (G2)]. Haploid and polyploid blood stages were distinguished based on Hoechst-fluorescence intensity and the values were used to calculate the number of nuclei per schizont. G2 is set at 8-45x the mean Hoechst-fluorescence value of ring forms, calculated from G1.

### Analysis of Oocyst and Sporozoite Production

For analysis of mosquito stages (oocysts and sporozoites), *Anopheles stephensi* mosquitoes were infected with day 14 gametocyte cultures using the standard membrane feeding assay (SMFA) (Ponnudurai et al., 1989; Marin-Mogollon et al., 2018). Oocysts and salivary gland sporozoites were counted at days 8–14 and 14–21 post feeding, respectively. Oocyst numbers and GFP/mCherry expression (see next section) in oocysts were determined at day 8 to 14 after feeding. Isolation of salivary gland sporozoites for counting numbers and expression of GFP/mCherry (see next section) was performed at day 14 to 21 after feeding. For counting sporozoites, salivary glands from 10 to 30 mosquitoes were dissected, collected in 100 μl of PBS and homogenized using a grinder. Sporozoites were counted using a Bürker cell counter using phase-contrast microscopy.

### Analysis of GFP and mCherry Expression by Fluorescence Microscopy

GFP- or mCherry-expression in different blood stages, oocysts and sporozoites was analyzed by standard fluorescence microscopy as previously described (Mogollon et al., 2016; Marin-Mogollon et al., 2019). In brief, for blood stages 200 μl samples of iRBC were collected from 10 ml cultures with a parasitemia between 4 and 10% and stained with the DNA-specific dye Hoechst-33342 by adding 4 μl of a 500 μM stock-solution (final concentration 10 μM) for 20 min at 37°C. Subsequently, 10 μl were placed on a microscope slide (mounted under a cover slip) and GFP/mCherry fluorescence of live infected RBC was analyzed using a Leica fluorescence MDR microscope (100x magnification). Pictures were recorded with a DC500 digital camera using ColourPro software and with the following exposure times: GFP 0.7 s; mCherry 1s; Hoechst 0.2 s; bright field 0.1 s (1x gain).

GFP/mCherry-expression in oocysts was determined at day 8 and 14 after infection of *A. stephensi* mosquitoes as described in the previous section. GFP/mCherry expression in sporozoites was determined at day 14 and 21 after infection of mosquitoes. Isolated sporozoites (see previous section) were pelleted by centrifugation (800 × g; 5 min). The pellet was suspended in [40 μl of PBS] and sporozoites stained with Hoechst-33342 (10 μM). Of this solution, 10 μl were placed on a microscopic slide (mounted under a cover slip) and live fluorescence of sporozoites was analyzed as described above. GFP/mCherry fluorescence of live oocysts and sporozoites was analyzed using a Leica fluorescence DMR microscope (100x magnification). Pictures were recorded with a DC500 digital camera microscope using ColourPro software and with the following exposure times: GFP 0.7 s; mCherry 1s; Hoechst 0.2 s; bright field 0.1 s (1x gain).

### Luciferase Assay

Luciferase expression in *mCh-Luc@sui1* and *mCh-Luc@40s* asexual blood-stages was determined as follows. Sample of asexual blood-stages (in triplicate) were prepared from blood-stage cultures with 1.0 × 10^6^ parasites per sample. Complete lysates (100 μl) were collected in a black 96-well plate (flat bottom) and luciferase activity was measured after adding 50 μl of Luciferase substrate (Luciferase Assay System Promega). Luciferase activity (in relative light units; RLU) was measured using the Glomax multi detection system Luminometer (Promega) and the Instinct software (Promega).

### Primary Human Hepatocyte Infection and Analyses

Liver segments from surgical patients were obtained 2 days prior to infection with sporozoites. Hepatocytes were isolated by perfusing the tissue with increasing concentrations of collagenase as described (Lecluyse and Alexandre, 2010). Viable cells were resuspended in complete William's B medium (William's E medium with Glutamax (Gibco, 32551-087), supplemented with 1%(v/v) insulin/transferrin/selenium (Gibco, 41400-045), 1%(v/v) sodium pyruvate (Gibco, 11360-036), 1% (v/v) MEM-NEAA (Gibco, 1140-035), 2.5 μg/ml Fungizone Antimycotic (Gibco, 15290-018), 200 Units/ml penicillin/streptomycin (Gibco, 15140-122), and 1.6 μM dexamethasone (Sigma Aldrich D4902-100MG), stained with trypan blue and counted. Approximately 62.000 cells were seeded (per 125 μl total volume) into each well of black 96 well plates (Falcon, 353219) coated with 0.056 mg/ml rat tail collagen I/well (Sigma Aldrich - 11179179-001). Hepatocytes were cultured at 37°C in an atmosphere of 5% CO_2_ with daily medium refreshment. A total of 62.000 sporozoites (MOI of 1:1) were added onto the hepatocytes 48 h post plating. The sporozoites were dissected in complete William's B media and then supplemented with 10% heat inactivated human serum (HIHS) upon addition to the well. The parasites were spun down onto the monolayer at 100 × g for 10 min with low brakes. The culture was incubated at 37°C in an atmosphere of 5% CO_2_ for a further 3 h, after which there was a medium refreshment with complete William's B media supplemented with 10% HIHS. The culture was cultured at 37°C in an atmosphere of 5% CO_2_ with daily medium refreshment (complete William's B with 10% HIHS). On Day 3, 5, 7, and 9, the cultures were imaged live using Zeiss LSM880 with Airyscan at 63x objective at 4x zoom. After live-imaging, the plates were fixed and an immunofluorescence assay was performed to stain for standard liver stage markers as described (Roth et al., 2018). Briefly, the culture is fixed in 4% PFA and blocked in 3% BSA. Mouse anti-EXP1, EXP2, GAPDH, and MSP1 were diluted 1:1000, 1:1000, 1:50.000, and 1:100 respectively. These antibodies (except MSP1) were acquired from the European malaria reagent repository: catalog numbers 5.1 (EXP-1), 7.7 (EXP2), and 7.2 (GAPDH). MSP1 was obtained from Sanaria, Catalog number AD233. Rabbit anti-HSP70 were diluted at 1:75. Presence of GFP was detected using polyclonal rabbit anti-GFP Alexa Fluor 488 (Thermofisher Cat#: A-21311) at 1:100 dilution. This antibody was applied with the secondary antibodies: Donkey anti-mouse 647 (Thermofisher Cat#: A31571) and goat anti-Rabbit 594 (Thermofisher Cat#: A11037) used at 1:200 dilution. Nuclear content was stained using DAPI (Thermofisher Cat#: D1306) used at 300 nM final concentration Confocal images were taken using Zeiss LSM880 with Airyscan on the 63x objective at 2x zoom. Tiles (9 × 9) of each well were taken using the Leica DM1600B on the 20x objective from which infection rates were calculated by counting the number of liver stages present and dividing it with the number of host cells present. Furthermore, 20 random tiles were selected for which the size of the liver stages (~100 liver stages) were measured using the region of interest (ROI) function of FIJI, an open sourced program designed by Schindelin et al. (2012).

## Results and Discussion

### *In silico* Identification of Constitutive and Strong Promoters for Reporter Expression

To select promoters for driving constitutive expression of reporters, we first searched for genes that are highly transcribed in multiple life cycle stages, specifically housekeeping genes that show strong expression, not only in blood stages but also in sporozoites and liver stages and whose transcript levels are similar or higher in comparison with the housekeeping genes *ef1*α (elongation factor 1-alpha; PF3D7_1357000) and *hsp70* (heat shock protein 70; PF3D7_0818900). Promoters of these latter two genes have been used previously to drive reporter expression in *P. falciparum* (Pf); however, reporter expression with the *ef1*α promoter in sporozoites and early liver stages is weak and reporter expression in liver stages with the *hsp70* promoter was not successful (see **Introduction section**). For analyses of transcript levels we used published genome-wide transcriptome data available from the PlasmoDB database (www.PlasmoDB.org) for *Pf* (see Table S2) (Otto et al., 2010; Lasonder et al., 2016; Zanghi et al., 2018) and from published transcriptome data of blood, mosquito and liver stages of *Plasmodium berghei* (Otto et al., 2014; Caldelari et al., 2019). In the absence of *Pf* transcriptome data of liver stages, we also analyzed transcriptome datasets of liver stages of two other human/primate *Plasmodium* species *Plasmodium vivax* and *Plasmodium cynomolgi* (Cubi et al., 2017; Voorberg-van der Wel et al., 2017; Gural et al., 2018). Based on analyses of these different datasets we selected two housekeeping genes involved in protein synthesis, i.e., translation initiation factor SUI1, putative (*sui1*, PF3D7_1243600) and 40S ribosomal protein S30 (*40s*, PF3D7_0219200). Both genes show high transcript levels in most life cycle stages (Table S2) with particularly *sui1* showing particularly high transcript abundance in sporozoites and liver stages of human/primate *Plasmodium* species.

### Analysis of Promoter Regions of the Housekeeping Genes *sui1* or *40s* to Express the Reporters GFP and mCherry-Luciferase in *P. falciparum* NF54

First we tested reporter expression using the *40s* and *sui1* promoters in blood stages and mosquito stages of the NF54 laboratory strain (*Pf* NF54) since this strain is well-characterized with respect to genome sequence and has been used for generation of transgenic parasites using CRISPR/Cas9 genetic modification (Mogollon et al., 2016; Marin-Mogollon et al., 2019). In these experiments we used GFP and the fusion protein mCherry-luciferase as reporter proteins. As a control promoter we included the *ef1*α promoter that has been used previously for reporter expression in *Pf*. For introduction of the different transgene expression cassettes into the genome we selected the *p47* gene (PF3D7_1346800) as target locus since this locus has been shown to be suitable for introduction of transgenes in *Pf* NF54 parasites without compromising blood stage development and development in *A. stephensi* mosquitoes (Vaughan et al., 2012; Vos et al., 2015; Lucantoni et al., 2016; Marin-Mogollon et al., 2019).

We used the recently developed two plasmid CRISPR/Cas9 method for generation of the transgenic *Pf* parasites without retaining a selectable marker in the genome (Mogollon et al., 2016). The Cas9 construct pLf0019, that contains the Cas9 expression cassette and a blasticidin (BSD) drug-selectable marker cassette (Mogollon et al., 2016), was used in combination with the donor DNA plasmids, that are based on the published plasmid pLf0047 (Marin-Mogollon et al., 2019) and contains the *p47* sgRNA, the *hdhfr-yfcu* drug selectable marker (SM) cassette, the two *p47* homology (targeting) regions and the transgene expression cassettes. Three different donor DNA constructs were generated, pLf0117, pLf0123, and pLf0128 that contain the mCherry-luciferase (mCherry-Luc) fusion gene under control of the *ef1*α*, sui1*, and *40s* promoters respectively. In addition, two DNA donor constructs were generated, pLf0116 and pLf0127 that contain the GFP gene under control of the *ef1*α and *40s* promoters, respectively (see the Materials and Methods section and Figure S1 for more details of the generation of the plasmids). These five plasmids are designed to introduce the transgene expression cassettes into the *p47* gene locus by double crossover homologous recombination (Figure 1A and Figure S2).

**Figure 2:**
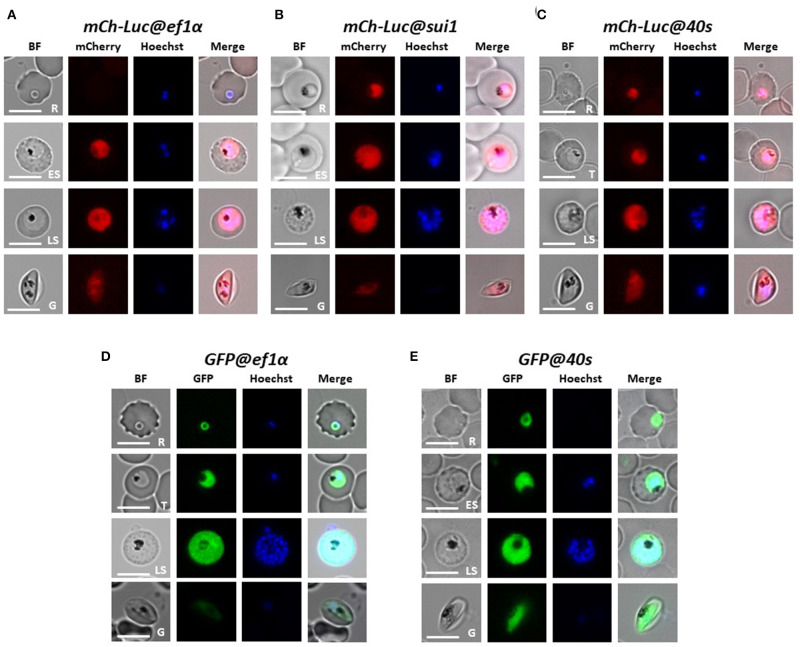
Expression of reporter proteins GFP or mCherry-luciferase in asexual blood stages and gametocytes of five transgenic *Pf* NF54 reporter lines. Representative fluorescence microscopy images of live *mCh-Luc@ef1*α*, mCh-Luc@sui1, mCh-Luc@40s* **(A–C)** and *GFP@ef1*α and *GFP@40s* **(D,E)** asexual blood-stages (R, rings; T, trophozoites; ES, early schizonts; LS, late schizonts) and stage III gametocyte (G). Nuclei were stained with Hoechst-33342. All pictures were recorded with standardized exposure/gain times to visualize differences in fluorescence intensity [GFP 0.7s; mCherry 1.0 s; Hoechst 0.2 s; bright field 0.1 s (1x gain)]. Bright field (BF). Scale bar, 7 μm.

Transfection of *Pf* NF54 parasites was performed by the method of pre-loading of erythrocytes with the plasmids, which are subsequently mixed with parasite-infected erythrocytes (Deitsch et al., 2001). After mixing, transfected parasites were selected by double positive selection using the drugs WR99210 and BSD. In independent experiments we obtained the following parasite populations, *mCh-Luc@ef1*α, *mCh-Luc@sui1, mCh-Luc@40s, GFP@ef1*α, and *GFP@40s*, expressing mCherry or GFP as determined by fluorescence microscopy analysis of mixed blood stages. For the *mCh-Luc@sui1* parasites, different clonal lines were obtained by limiting dilution. For *mCh-Luc@ef1*α, *mCh-Luc@40s* line, mCherry-positive cells were collected by flow cytometry (FACS) sorting using mixed blood stages, resulting in parasite populations in which more than 90% of blood stages were mCherry-positive (determined by fluorescence microscopy; Figure 2). Diagnostic PCR analyses on genomic DNA of the three mCherry-Luc lines indicate integration of the transgene-expression cassettes into the *p47* locus (Figure 1B). Southern analysis confirmed double crossover integration in parasites of these three lines and absence of episomal donor plasmids (Figure 1C). The *GFP@ef1*α and *GFP@40s* parasite populations were not cloned or enriched for transgenic parasites by FACS-sorting. In these populations more than 90% of mixed blood stages were GFP-positive (determined by fluorescence microscopy; Figure 2). Diagnostic PCR on genomic DNA of these two GFP lines indicates integration of the transgene expression cassettes into the *p47* locus (Figure S2). In addition, PCR amplification of the WT *p47* locus indicates the presence of WT parasites as was expected based on the presence of GFP-negative parasites in mixed blood stage populations of these two lines.

**Figure 3:**
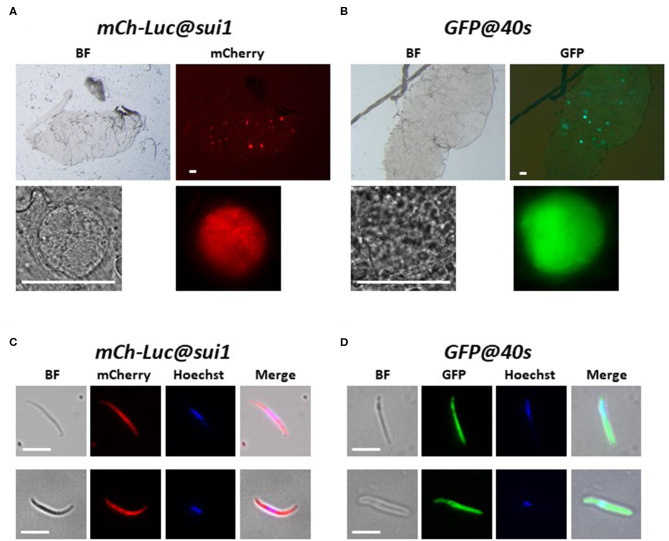
Expression of reporter proteins in oocysts and sporozoites of the transgenic *Pf* NF54 reporter lines *mCherry-luc@sui1* and *GFP@40s*. **(A,B)** Representative fluorescence microscopy images of live oocysts of *mCherry-Luc@sui1* and G*FP@40s* parasites in *A. stephensi* mosquitoes (day 12 after infection). Upper panel: oocysts in complete midgut and representative oocyst in lower panel. Bright field (BF). Scale bar, 40 μm. **(C,D)** Representative fluorescence microscopy images of live salivary gland sporozoites of *mCherry-Luc@sui1* and *GFP@40s* (day 21 after infection). Nuclei were stained with Hoechst33342. All pictures were recorded with standardized exposure/gain times to visualize differences in fluorescence intensity [GFP 0.7s: mCherry 1.0 s; Hoechst 0.2 s; bright field 0.1 s (1x gain)]. Bright field (BF). Scale bar, 7 μm.

We examined mCherry and GFP expression in blood stages and mosquito stages of the various transgenic lines. The different asexual blood stages (trophozoites, schizonts) and gametocytes (stage III) of all five lines expressed mCherry or GFP (Figure 2). In addition, we confirmed expression of luciferase in blood stages of two lines with the mCherry-luciferase fusion protein (Figure S3). We next passaged two of the lines, *mCh-Luc@sui1* and *GFP@40s* through *A. stephensi* mosquitoes. Oocysts and salivary gland sporozoites of both lines were mCherry- and GFP-positive, respectively (Figure 3), showing that both the *sui1* and *40s* promoter drive expression in mosquito stages. Combined these analyses show that the two selected promoter regions of the housekeeping genes, *sui1* and *40s*, are able to drive expression of reporter genes and both promoters drive expression in all blood stages and mosquito stages examined. Based on fluorescence intensity of these life cycle stages, the level of reporter expression with these two novel promoters appears to be similar or higher compared to reporter expression with the *ef1*α promoter (see also the next section describing transgenic *Pf* NF135 parasites expressing GFP using the three promoters).

### Generation and Genotyping of *P. falciparum* Transgenic NF135 Lines Expressing GFP Under Control of the *sui1* and *40s* Promoters

*Pf* NF135 parasites (*Pf* NF135) have a higher invasion rate of hepatocyte *in vitro* and produce more liver merozoites per infected hepatocyte as compared to *Pf* NF54 parasites (McCall et al., 2017). Transgenic *Pf* NF135 parasites with high reporter expression in liver stages may therefore be valuable tools for liver stage analyses. Since both the *sui1* and *40s* genes are highly transcribed in liver stages of the rodent parasite *P. berghei* and the human/primate *Plasmodium* species *P. vivax* and *P. cynomolgi* (Table S2), we generated two transgenic *Pf* NF135 lines expressing GFP under control of either the *sui1* or the *40s* promoter. As a control line, we also generated a *Pf* NF135 line expressing GFP under control of the *ef1*α promoter.

**Figure 4:**
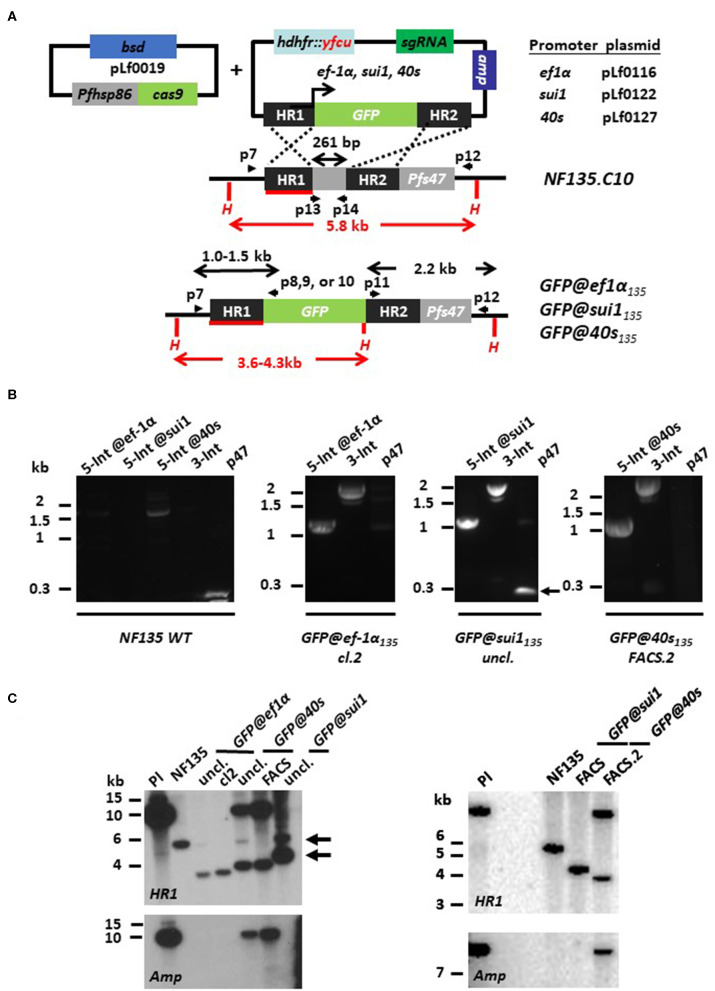
Generation of *P. falciparum* NF135 reporter lines expressing GFP under control of *ef1*α, *sui1* or *40s* promoter. **(A)** Schematic representation of the Cas9 (pLf0019) and sgRNA/donor (pL0116, pLf0122, pLf0127) constructs used to introduce the GFP expression cassette into the *P. falciparum* NF135 *p47* gene locus. The GFP gene is under the control of the promoter of the *ef1*α, *sui1*, or *40s* genes. The *p47* homology regions (HR1, HR2) used to introduce the donor DNA (i.e., the GFP expression cassette), location of primers (p), sizes of restriction fragments (H: *Hpa*I; in red) and PCR amplicons (in black) are indicated. Primer sequences (shown in black and bold) are shown in Table S1. WT, wild type; bsd, blasticidin selectable marker (SM); *hdhfr::yfcu*—SM in donor plasmid. **(B)** Diagnostic PCR confirms the correct 5′ integration into the genome of *GFP@ef1*α_135_*, GFP@sui1*_135_*, or GFP@40s*_135_ (5-Int; primers p7/p8 for *ef1*α 1,009 bp, p7/p9 for *sui1* 1,106 bp, p7/p10 for *40s* 1,087 bp) and correct 3′ integration (3-Int; primers p11/p12; 2,188 bp). In addition, it shows absence of the *p47* gene in *GFP@ef1*α_135_ clone 2 and in the FACS sorted line of *GFP@40s*_135_ (*p47* primers p13/p14; 216 bp). Primer locations and product sizes are shown in **(A)** and primer sequences in Table S1. The arrow indicates PCR product of WT *p47* gene amplified by p13/p14 primers. The weak 1.5 kb band with the 5-Int@40s-primers is a non-specific fragment which is only present in WT with and not in the transgenic lines with the GFP cassette. **(C)** Southern analysis of *Hpa*I restricted DNA to confirm correct integration of the plasmids in the three transgenic lines. Digested DNA was hybridized with a probe targeting the homology region 1 of *p47* [HR1; shown in red; see **(A)**] and with a probe recognizing ampicillin (Amp; fragment of ~10 kb). Left panel: WT NF135 shows the expected 5.8 kb fragment. In both uncloned and clone 2 of *GFP@ef1*α_135_ parasites the expected 3.6 kb fragment is present after double crossover (DXO) integration. In uncloned and FACS-sorted populations of *GFP@40s*_135_ parasites two fragments with the expected sizes of 3.8 kb and ~10 kb of single crossover integration are present whereas in the uncloned *GFP@sui1* the expected fragment of 4.3 kb of double crossover integration and the fragment of 5.8 kb of WT is present (see arrows). Hybridization with the Amp probe shows the single crossover events in the GFP@40s populations. Right panel: After additional FACS sorting of GFP-positive parasites Southern analysis shows the expected 5.8 kb fragment in *GFP@sui1* of double crossover integration whereas in *GFP@40s* the two fragments are present with the expected sizes of 3.8 kb and ~10 kb of single crossover integration. The presence of SXO parasites in *GFP@40s*_135_ is confirmed by hybridization with a probe recognizing ampicillin (Amp; fragment of ~10 kb).

We first compared the published genome sequences of the promoter regions of *ef1*α*, sui1*, and *40s* in the *Pf* NF54 genome (www.GeneDB.org) and the *Pf* NF135 genome (Moser et al., 2019). The promoter regions selected from the *Pf* NF54 (see above) were almost identical (99–100% similarity) to those of *Pf* NF135. Also, the sequence of the *p47* target regions were more than 99% similar and the *p47* guide RNA sequence was identical in both lines (data not shown). Based on the sequence similarities, we decided to use the same sequences/basic plasmids for generation of the donor DNA constructs to create the *Pf* NF135 reporter lines as were used for the *Pf* NF54 reporter lines (see above). We used the final DNA donor plasmids pLf0116 and pLf0127 for introduction into the genome of the *ef1*α-GFP and *40s*-GFP expression cassettes (as described in the previous section). For introduction of the *sui1*-GFP expression cassette, we generated the final donor DNA plasmid pLf0122 (Figure 4A; see the Materials and Methods section and Figure S1 for more details of the generation of the plasmid). Transfection with the final donor DNA constructs and the Cas9 construct pLf0019, followed by positive drug selection (pyrimethamine and BSD) and negative selection (5-FC) was performed as described in the Materials and Methods section and resulted in selection of the three parasite populations *GFP@ef1*α_NF135_, *GFP@40s*_NF135_, and *GFP@sui1*_NF135_ expressing GFP as determined by fluorescence microscopy analysis of mixed blood stages. For the *GFP@40s*_NF135_ and *GFP@sui1*_NF135_ populations we performed two rounds of FACS-sorting using mixed blood stages to enrich for transgenic GFP-positive parasites, resulting in parasite populations where more than 99% of the blood stages were GFP-positive. From the *GFP@ef1*α _NF135_ population we obtained a GFP-expressing clone (clone 2) by the method of limiting dilution. For all three lines we performed a PCR analysis that distinguishes between *Pf* NF54 and *Pf* NF135 parasites. This analysis confirmed the *Pf* NF135 background of the three GFP-expressing lines (Figure S4).

Diagnostic PCR on genomic DNA of the three GFP lines indicates correct integration of the transgene-expression cassettes into the *p47* locus (Figure 4B). We could not detect a PCR fragment of the *p47* open reading frame in the FACS-sorted *GFP@40s*_NF135_ and *GFP@ef1*α _NF135_ clone2, indicating homogenous and pure populations of transgenic parasites with the GFP-expression cassettes integrated into the *p47* locus. Southern analysis confirmed that the *GFP@ef1*α _NF135_ clone2 consists of a pure population of transgenic parasites containing the GFP-expression cassette integrated by double crossover recombination into the *p47* locus (Figure 4C). Southern analysis indicates that both FACS-sorted *GFP@40s*_NF135_ and *GFP@sui1*_NF135_ populations consist of transgenic parasites with the GFP-expression cassette integrated either by double crossover (*GFP@sui1*_NF135_) or single crossover recombination (*GFP@40s*_NF135_) (Figure 4C). This Southern analysis cannot distinguish between episomal plasmid and single crossover integration. However, in these parasites the 12 kb fragment it is most likely integrated plasmid since episomal plasmid is usually lost during in cultures without drug-selection for prolonged periods.

### Growth Characteristics of Blood and Mosquito Stages of Transgenic *Pf*NF135 Lines Expressing GFP by the *sui1* and *40s* Promoters

We first examined growth characteristics of blood stages and mosquito stages of the three transgenic GFP-expressing *Pf* NF135 lines. Though it has been shown that disruption of the *p47* gene locus in *Pf* NF54 parasites does not compromise parasite development in blood stages and liver stages as well as in *A. stephensi* mosquitoes (Talman et al., 2010; Vaughan et al., 2012; Marin-Mogollon et al., 2019), the effect of *p47* disruption in *Pf* NF135 parasite is unknown. For use of these reporter lines in future studies it is important that they retain growth and development kinetics of the parental *Pf* NF135 line.

*In vitro* growth of asexual blood stages of the three lines was comparable to the growth of asexual blood-stages of the parent WT *Pf* NF135 line (Figure S5) and they produced WT-comparable numbers of mature stage V gametocytes in standardized gametocyte cultures (Table 1). We next passaged the three lines through *A. stephensi* mosquitoes and analyzed production of oocysts and salivary gland sporozoites. All three lines produced oocysts and sporozoites and oocyst and sporozoite numbers were in the same range as WT *Pf* NF135 parasites (Table 1). Since we have passaged WT *Pf* NF135 only once through mosquitoes, we compared oocysts and sporozoite production also with those of *Pf* NF54 parasites, which have been frequently transmitted through mosquitoes in our laboratory. Oocyst and sporozoite production of the three transgenic lines (mean of 5–15 oocyst per mosquito) is lower than those of *Pf* NF54 parasites (mean of 21–67) which is in agreement to published observations on mosquito transmission of *Pf* NF135 (Teirlinck et al., 2013). Combined, these results indicate that the three transgenic lines have growth characteristics of blood stages and mosquito stages that are comparable to the parent *Pf* NF135 line and all three lines produce salivary gland sporozoites, which will permit further analyses of sporozoite infectivity and liver stage development (see below). These results also demonstrate that the *p47* gene can be used as a target locus for introduction of transgenes in *Pf* NF135 parasites.

**Table 1:** Gametocyte, oocyst and sporozoite production of three transgenic *Pf* NF135 reporter lines.

**Lines**	**Stage V Gametocytemia^a^**	**No. of oocysts^b^**	**No. of sporozoites ( × 10^3^)^c^**
	**Average (SD)**	**Average (SD)**	**Average (SD)**
*NF135 WT*	0.9	5.4	8.0
	(1 exp.)	(1 exp.)	(1 exp.)
*GFP@ef-1α_*NF*135_*	0.7 (0.3)	15 (7.4)	16 (4.0)
	(3 exp.)	(2 exp.)	(3 exp.)
*GFP@sui1_*NF*135_*	0.5 (0.3)	13 (0.2)	11 (2.4)
	(3 exp.)	(2 exp.)	(2 exp.)
*GFP@40s_*NF*135_*	0.6 (0.4)	9.2 (1.3)	11 (3.3)
	(4 exp.)	(3 exp.)	(2 exp.)
*NF54 WT*	0.8–1.2 (0.2)	21–67 (17)	8–45 (19)
	(4 exp.)	(8 exp.)	(4 exp.)

a *Percentage of stage V gametocytes (per 100 red blood cells) in day 14 cultures*.

b *Mean number of oocyst per mosquito at day 7–12 after feeding (10–30 mosquitoes per exp.)*.

c *Mean number of salivary gland sporozoites per mosquito at day 21 after feeding (20–30 mosquitoes per exp.)*.

### Analysis of GFP-Expression Throughout the Complete Life Cycle of Transgenic *Pf*NF135 Lines Expressing GFP by the *sui1* and *40s* Promoters

**Figure 5:**
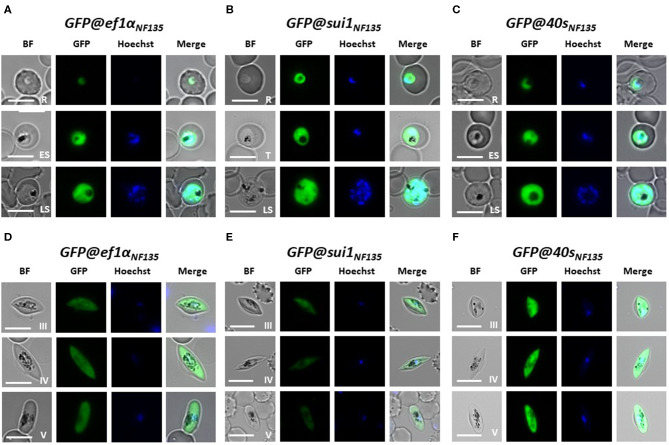
Expression of GFP in asexual blood stages and gametocytes of three transgenic *Pf* NF135 reporter lines. Representative fluorescence microscopy images of live *GFP@ef1*α_*NF*135_*, GFP@sui1*_*NF*135_, and *GFP@40s*_*NF*135_ asexual blood-stages **(A–C)** and gametocytes **(D–F)**. R, rings; T, trophozoites; S, schizonts; G, gametocytes stage III, IV, and V. Nuclei were stained with Hoechst-33342. All pictures were recorded with standardized exposure/gain times to visualize differences in fluorescence intensity [GFP 0.7 s; Hoechst 0.2 s; bright field 0.1 s (1x gain)]. Bright field (BF) Scale bar, 7 μm.

**Figure 6:**
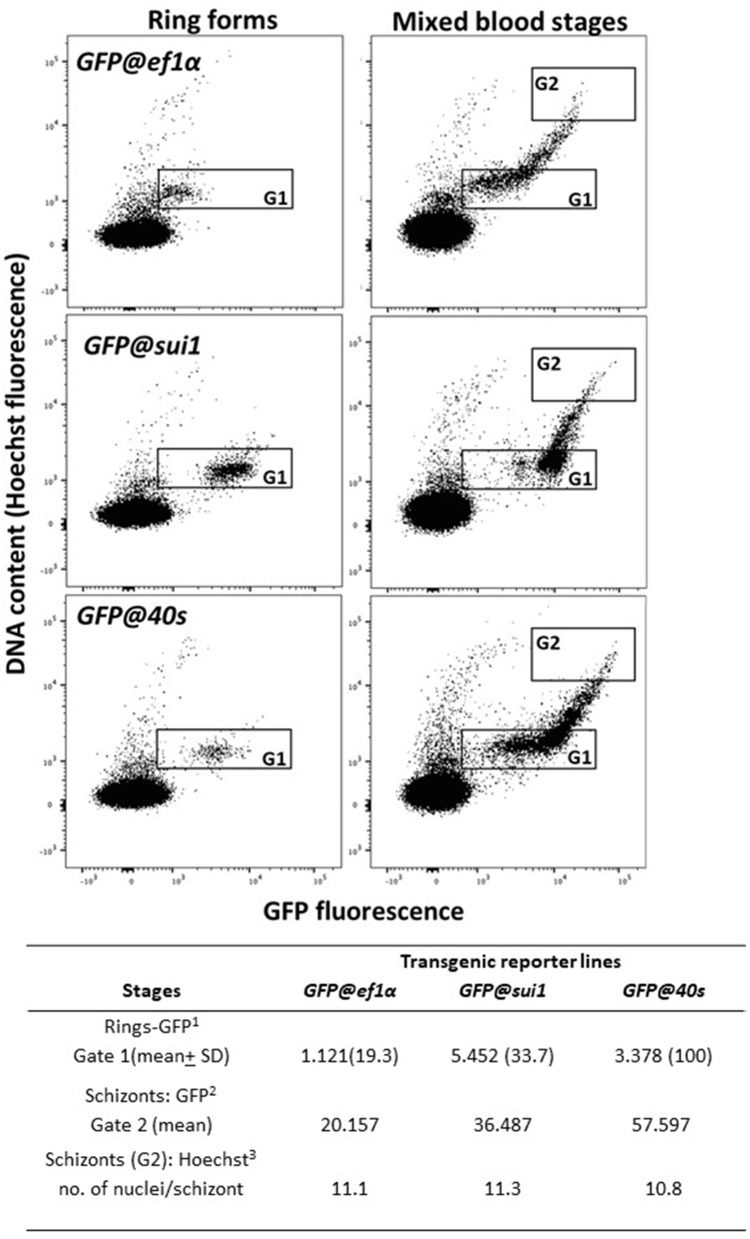
GFP and Hoechst33258 fluorescence intensities of ring forms and schizonts in transgenic PfNF135 reporter lines based on FACS analysis. Fluorescence intensity of rings (**left** panel) and mixed blood stages (**right** panel) as determined by flow cytometry. Infected red blood cells (RBC) were stained with the DNA-specific dye Hoechst33258 to distinguish infected RBC from uninfected RBC. Left panel: Gate 1: synchronized ring forms; Right panels: Gate 2 (G2): schizonts. The gate is set at 8-45x the mean Hoechst fluorescence value of ring forms. The panels show dot plots of both Hoechst fluorescence intensity (DNA content; x-axis) and GFP fluorescence intensity (y-axis). In the Table the mean values are shown of the GFP-intensity of ring forms in G1 and schizonts in G2. ^1^GFP fluorescence intensity of (ring) infected red blood cells in Gate 1 ^2^GFP fluorescence intensity of (schizont) infected red blood cells in Gate 2. ^3^The number of nuclei is calculated by dividing the mean Hoechst fluorescence intensity of schizonts in Gate 2 by the mean Hoechst fluorescence intensity of rings in Gate 1.

We examined GFP expression in the different reporter lines during blood stage development by fluorescence microscopy. Expression of GFP was detectable throughout blood stage development, from ring forms to mature schizonts of *GFP@40s*_NF135_, *GFP@sui1*_NF135_ and the control line *GFP@ef1*α_NF135_ (Figures 5A–C). Gametocytes were also GFP-positive with gametocytes of the *GFP@40s*_NF135_ line showing brightest fluorescence (Figures 5D–F). The GFP-expression in all blood stages, including gametocytes, is in agreement with data on transcription of the three genes from which the promoters were used (Table S2). Next, we more precisely compared the GFP-fluorescence intensity of the different lines by examining mixed blood stages by flow cytometry as described previously for GFP-expressing *Pf* NF54 reporter lines (Mogollon et al., 2016). In this analysis the different blood stages are distinguished based on their fluorescence intensity (DNA content) after staining with the DNA-specific dye Hoechst-33258. In all three lines GFP-fluorescence intensity increased during the period of development of rings into mature schizonts (Figure 6). Haploid ring forms (1N) of both *GFP@40s*_NF135_ and *GFP@sui1*_NF135_ showed 3–5 times higher fluorescence intensity than *GFP@ef1*α _NF135_ ring forms with highest intensity of *GFP@sui1*_NF135_ rings. *GFP@40s*_NF135_ schizonts (mean of 11 nuclei) showed highest fluorescence intensity with 1.6–2.8 times the intensity of *GFP@sui1*_NF135_ and *GFP@ef1*α_NF135_ schizonts, respectively.

**Figure 7:**
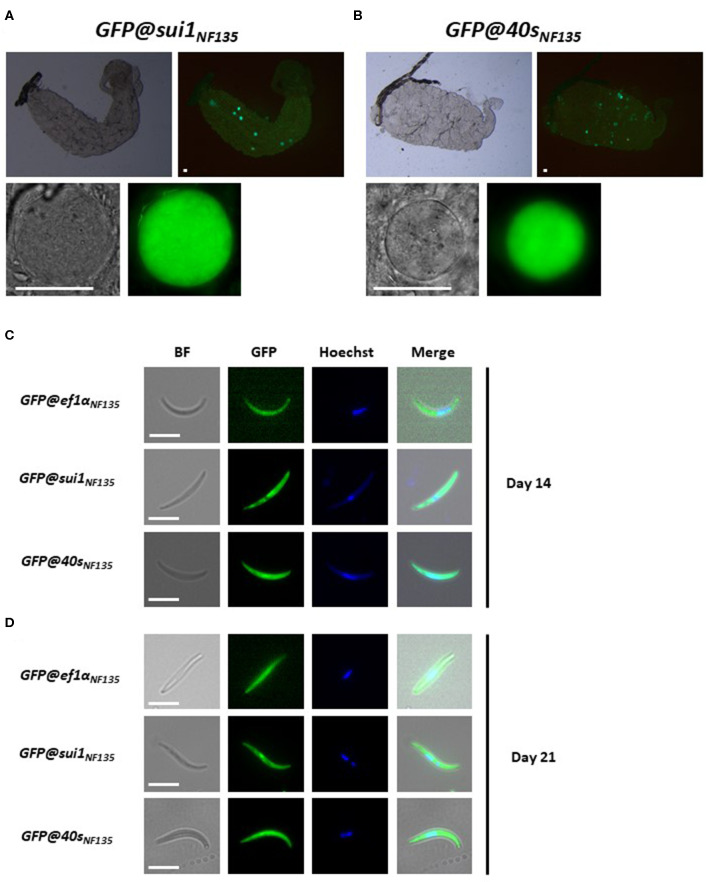
Expression of GFP in oocysts and sporozoites of the transgenic *Pf* NF135 reporter lines. **(A,B)** Representative fluorescence microscopy images of live oocysts of *GFP@sui1* and G*FP@40s* parasites in *A. stephensi* mosquitoes. Upper panel: oocysts in complete midgut and representative oocyst in lower panel. Scale bar, 40 μm. **(C,D)** Representative GFP-fluorescence microscopy images of live salivary gland sporozoites of *GFP@ef1*α_*NF*135_*, GFP@sui1*_*NF*135_, and *GFP@40s*_*NF*135_ collected at day 14 and 21 after infection. Nuclei were stained with Hoechst33342. Bright field (BF). Scale bar, 7 μm.

We next examined GFP expression in oocysts and sporozoites, collected from *A. stephensi* mosquitoes, which were fed with gametocytes of the three transgenic lines using the standard membrane feeding assay. Like oocysts and sporozoites of *Pf* NF54 lines that expressed reporters by the *sui1* and *40s* promoter, oocyst and sporozoites of both *GFP@sui1*_NF135_ and *GFP@40s*_NF135_ expressed GFP and these life cycle stages were strongly GFP-positive (Figure 7).

**Figure 8:**
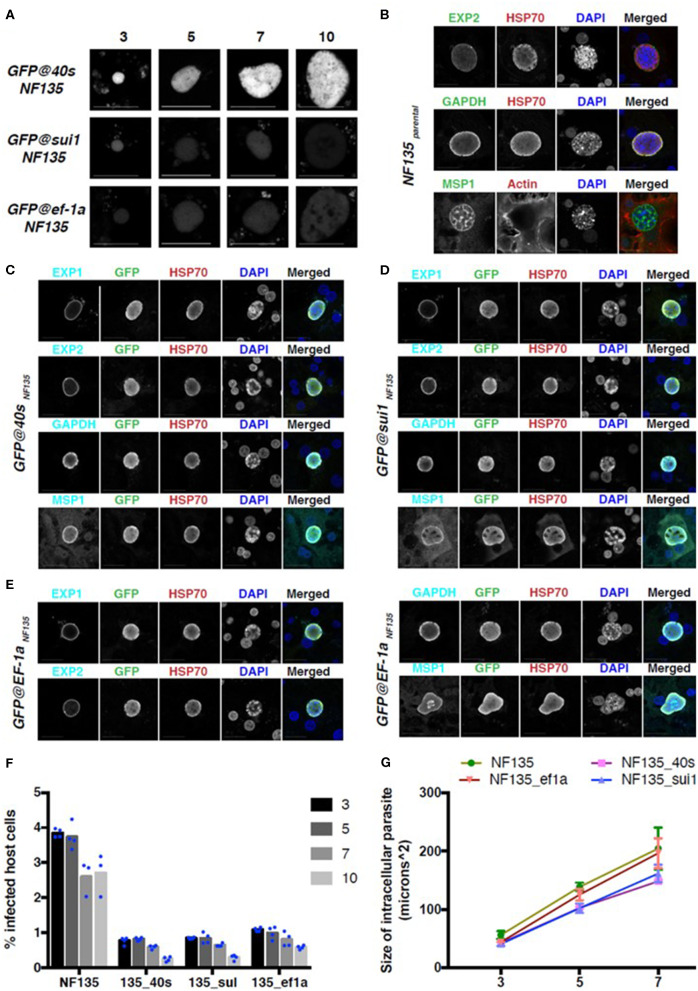
GFP expression and development in three NF135 reporter lines in the liver stage. **(A)** Representative GFP-fluorescence microscopy images of liver stage parasites of *GFP@ef1*α_*NF*135_, *GFP@sui1*_*NF*135_, and *GFP@40s*_*NF*135_ at different days after adding sporozoites to primary human hepatocytes. Scale bar, 25 μm. **(B–E**) Representative confocal microscopy images of liver stage parasites on day 7 for *Pf* NF135_parental_ **(B)**, *GFP@40s*_*NF*135_ **(C)**, *GFP@sui1*_*NF*135_ **(D)**, and *GFP@ef1*α_*NF*135_ **(E)**. Parasites were stained with antibodies against the *P. falciparum* proteins EXP1, EXP2, GAPDH, and MSP1 (cyan), HSP70 (red) and against GFP (green). DNA was stained with DAPI (blue). Scale bar, 25 μm. **(F)** The percentage of host cells infected with *Pf* NF135, *GFP@40s*_*NF*135_*, GFP@sui1*_*NF*135_, and *GFP@ef1*α_*NF*135_ on day 3, 5, 7, and 10 post invasion. Each dot represents one well from one experiment. **(G)** The size of intracellular parasites on day 3, 5, or 7 post invasion for *Pf* NF135, *GFP@40s*_*NF*135_*, GFP@sui1*_*NF*135_, and *GFP@ef1*α_*NF*135_. At least 50 cells were measured per well, resulting in a total of ~150 cells (3 wells per time point). The average of medians from triplicates and S.D. are shown. Dunnett's multiple comparison test was performed: *p*-value between *Pf* NF135 and *GFP@40s*_*NF*135_ is 0.0023; *Pf* NF135 and *GFP@sui1*_*NF*135_ is 0.0045; and *Pf* NF135 WT and *GFP@ef1*α_*NF*135_ is 0.3153.

Finally, we examined reporter expression during liver stage development of the three *Pf* NF135 reporter lines in primary human hepatocytes. Primary human hepatocytes (PHH) were infected with 5 × 10^4^ sporozoites of the *GFP@40s*_NF135_, *GFP@sui1*_NF135_, and *GFP@ef1*α_NF135_ per well of a 96-wells plate. Live imaging for GFP expression was performed using confocal fluorescence microscopy on day 3, 5, 7, and 10 (Figure 8A). Both *GFP@40s*_NF135_ and *GFP@sui1*_NF135_ had increased GFP expression on day 3 compared to *GFP@ef1*α _NF135_. While the GFP expression levels for *GFP@sui1*_NF135_ decreased to similar levels compared to *GFP@ef1*α_NF135_ as liver stage development progressed, it remained strong for *GFP@40s*_NF135_.

To check for proper development of the reporter lines in PHH, protein expression of standard liver stage markers such as Exp1 (PF3D7_1121600), Exp2 (PF3D7_1471100), GAPDH (PF3D7_1462800), and the late stage marker MSP1 (PF3D7_0930300) were examined on day 7 post invasion and compared with expression in livers stages of wild type *Pf* NF135 (Figures 8B–E). Additionally, to examine the infection rate of and maintenance of developing liver stages of the reporter lines in PHH compared to the *Pf* NF135 WT, the number of infected host cells were calculated using high content fluorescence microscopy after staining with anti-*Pf* HSP70 (PF3D7_0818900) on Day 3, 5, 7, and 10 (Figure 8F). Unexpectedly, all three *Pf* NF135 reporter lines infected significantly fewer PHH on all 4 time points examined (Figure 8F). Furthermore, intracellular *GFP@40s*_NF135_ and *GFP@sui1*_NF135_ parasites were significantly smaller than the *Pf* NF135 parental line and were more comparable in size to *Pf* NF54 when examined on day 5 and day7 post invasion (Figure 8G and Figure S6). The reason for the lower infectivity of sporozoites of the *Pf* NF135 reporter lines is unclear. It is possible that high expression of the reporter protein GFP slightly affects both sporozoite infectivity and development of liver stages. However, in previous experiments with *Pf* NF135 parasites a relatively large variation has been observed in size of liver stages, measured at day 6 where the size of *Pf* NF54 livers stage schizonts (6.3–16.1 μm) overlap with that of *Pf* NF135 schizonts (12.5–28.6) (McCall et al., 2017). A similar variation and overlap was found in sporozoite infectivity between the two strains as determined by measuring infected hepatocytes at day 2 and 5 after adding sporozoites to freshly isolated human hepatocytes in 3 independent experiments (McCall et al., 2017). Therefore, it would be important to confirm in future experiments with these reporter lines whether the lower infectivity is a characteristic of the reporter lines or is due to inter-experimental variation.

## Concluding Remarks

Combined our analyses identified novel promoter regions of two housekeeping genes, *sui1* and *40s*, that can drive strong expression of reporter proteins throughout the complete *Pf* life cycle. These promoter sequences are conserved in sequence between *Pf* NF54 and *Pf* NF135.C10 parasites and, as expected, we found that the promoters selected and derived from the *Pf* NF54 parasite genome drive expression of reporters in *Pf* NF135 parasites with a similar pattern as in *Pf* NF54 parasites. We report for the first time the generation of transgenic parasites in Cambodian *Pf* NF135 parasites and show that CRISPR/Cas9 constructs can be used that are based on constructs containing only *Pf* NF54 genome sequences. In addition, our analyses demonstrate that, like in *Pf* NF54 parasites, the *p47* gene locus can be used as a silent target locus for introducing transgenes in *Pf* NF135 parasites without reducing oocyst and sporozoite production in *A. stephensi* mosquitoes. We found that specifically the *40s* promoter drives high expression in all life cycle stages with higher expression in gametocytes and liver stages compared to the *sui1* promoter. In view of the high-level reporter expression in liver stages, the *GFP@40s* reporter lines may be beneficial for further analysis of liver stage development, both for analyses *in vitro* in primary hepatocytes and *in vivo* in humanized mice, for example in screening assays for novel drugs/small molecule inhibitors. In addition, it may also enhance collection of infected hepatocytes by flow cytometry/FACS sorting, as has been performed in rodent malaria parasites, which will permit genome wide analyses of *P. falciparum* liver stage such as proteomics, transcriptomic and lipidomics. Such larger scale analyses are still lacking for *P. falciparum* liver stages but are essential for a better understanding in this part of the *Plasmodium* life cycle (Vaughan and Kappe, 2017).

## Data Availability Statement

All datasets generated for this study are included in the article/Supplementary Material.

## Ethics Statement

Primary human liver cells were freshly isolated from remnant surgical material. The samples are anonymized and general approval for use of remnant surgical material was granted in accordance to the Dutch ethical legislation as described in the Medical Research (Human Subjects) Act, and confirmed by the Committee on Research involving Human Subjects, in the region of Arnhem-Nijmegen, the Netherlands.

## Author's Note

This paper is dedicated to the memory of our friend and colleague, SMK, who recently passed away.

## Author Contributions

SM, CJ, and SMK came up with the study concept and design. SM, AY, FG, CM-M, YM, TI, SKK, JR, SC-M, AS, and YW acquired the data. SM, AY, BF-F, CJ, and SMK conducted analysis and interpretation of the data. SM, AY, and CJ wrote the draft of the manuscript. SM, AY, BF-F, and CJ critically revised the manuscript for important intellectual content. G-JG, BF-F, AH, and RS provided technical and/or material support. CJ and SMK supervised the study. All authors reviewed the manuscript.

## Conflict of Interest

RS was employed by the company TropIQ Health Sciences. The remaining authors declare that the research was conducted in the absence of any commercial or financial relationships that could be construed as a potential conflict of interest.

## Supplementary Material

Figure S1 Maps of DNA vectors used in this study. Vector maps of DNA plasmids used to generate the different transgenic *Pf* NF54 and *Pf* NF135 expressing either GFP or mCherry-Luciferase under control of the of *ef1*α*, sui1*, and *40s* promoters. See Materials and Methods section for description and details of the generation of these plasmids.

Figure S2 Generation of *P. falciparum* NF54 reporter lines expressing GFP under control of *ef1*α or *40s* promoter. **(A)** Schematic representation of the Cas9 (pLf0019) and donor DNA plasmids (pL0116, pLf0127) constructs used to introduce the GFP expression cassette into the *Pf* NF54 *p47* gene locus. The GFP gene is under the control of the promoter of the *ef1*α or *40s* gene. The *p47* homology regions (HR1, HR2) used to introduce the donor DNA (i.e., the GFP expression cassette), location of primers (p), and PCR amplicons (in black) are indicated. Primer sequences (shown in black and bold) are shown in Table S1. WT, wild type; bsd, blasticidin selectable marker hdhfr::yfcu—SM in donor plasmid. **(B)** Diagnostic PCR confirms the correct 5′ integration of the plasmids into the genome of *GFP@ef1*α and *mCh-Luc@40s* parasites (5-Int; primers p7/p8 for *ef1*α 1,009 bp, p7/p10 for *40s* 1,087 bp) and correct 3′ integration (3-Int; primers p11/p12; 2,188 bp). Primer locations and product sizes are shown in **(A)** and primer sequences in Table S1. The arrow indicates PCR product of WT *p47* gene amplified by p13/p14 primers (216 bp).

Figure S3 Luciferase expression in blood stages of *mCh-Luc@sui1* and *mCh-Luc@40s*. Luciferase activity (RLU, relative light units) in asexual blood stages of *mCh-Luc@sui1 (cl.3), mCh-Luc@40s (FACS)*, and wild type *Pf* NF54 parasites (WT). The mean luminescence value of triplicate samples is shown (1.0 × 10^6^ blood stages per sample); error bars represent the standard deviation.

Figure S4 Diagnostic PCR on genomic DNA of *GFP@ef1*α_*NF*135_*, GFP@sui1*_*NF*135_, and *GFP@40s*_*NF*135_ to distinguish *Pf* NF54 and *Pf* NF135. Diagnostic PCR confirms the *Pf* NF135 genetic background of the *GFP@ef1*α_*NF*135_*, GFP@sui1*_*NF*135_, and *GFP@40s*_*NF*135_ lines. PCR amplification of the *glurp* gene (primers p15/p16) and the *msp2* gene (primers p17/18) resulting in the expected PCR fragments of ~700 and ~900 bp, respectively in NF135 strain.

Figure S5 Characterization of the growth kinetics of asexual blood stages of *GFP@ef1*α_*NF*135_*, GFP@sui1*_*NF*135_, and *GFP@40s*_*NF*135_. Growth of asexual blood-stages of *GFP@ef1*α clone2*, GFP@sui1* (FACS sorted), and *GFP@40s* (FACS2 sorted) and wild type (WT) *Pf* NF135. Parasitemia (mean and S.D of three independent cultures) is shown during a 3-day culture period (in the semi-automated culture system). Cultures were initiated with a parasitemia of 0.1%.

Figure S6 The size of three NF135 reporter lines in the fresh primary human hepatocytes **(A)**. Sizes of *Pf* NF135 **(A)**, *GFP@40s*_*NF*135_ **(B)**, *GFP@sui1*_*NF*135_**(C)** and *GFP@ef1*α_*NF*135_ **(D)** intracellular parasites for three different wells at each time point. Each box plot is made up of measurements of at least 50 intracellular parasites. Horizontal line within each box plot represents the median and the dots shows value outside the 5–95% confidence interval.

Table S1 Primers used in this study.

Table S2 RNAseq data (RPKM values) of different Plasmodium housekeeping genes in multiple life cycle stages obtained from published genome-wide transcription studies.
