# Supplementary figures and images for: Generation of Novel Plasmodium falciparum NF135 and NF54 Lines Expressing Fluorescent Reporter Proteins Under the Control of Strong and Constitutive Promoters
Source: Front Cell Infect Microbiol. 2020 Jun 10;10:270. doi: 10.3389/fcimb.2020.00270 (PMC7298075; doi:10.3389/fcimb.2020.00270)

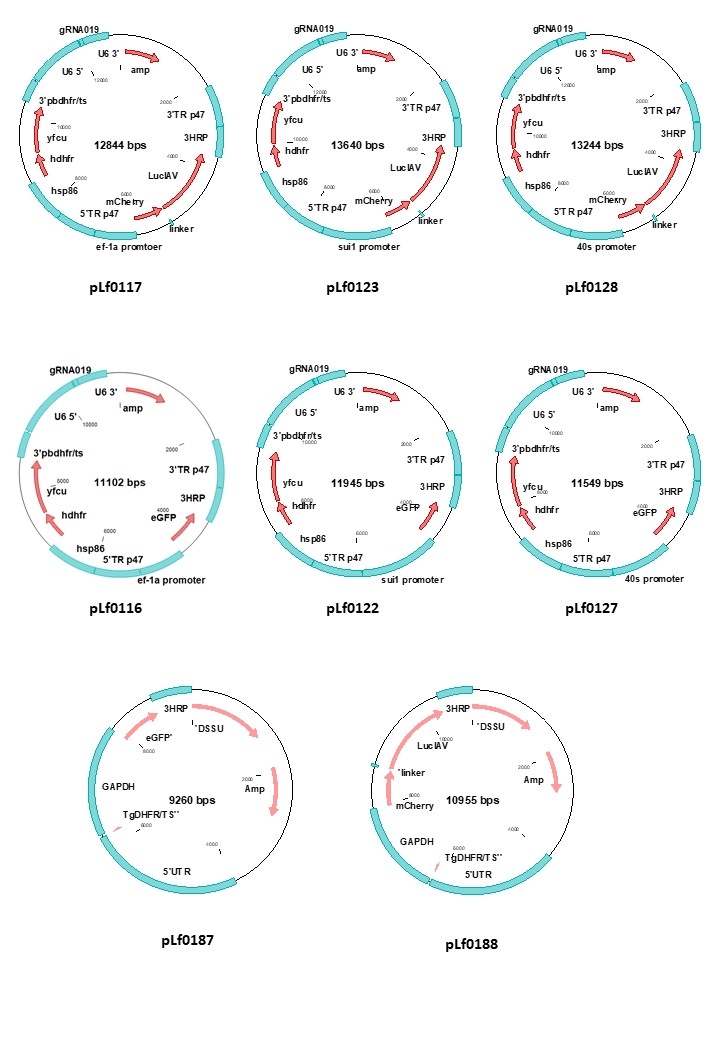

Supplement: Figure S1 — Maps of DNA vectors used in this study. Vector maps of DNA plasmids used to generate the different transgenic Pf NF54 and Pf NF135 expressing either GFP or mCherry-Luciferase under control of the of ef1α, sui1, and 40s promoters. See Materials and Methods section for description and details of the generation of these plasmids. [file Image_1.JPEG]

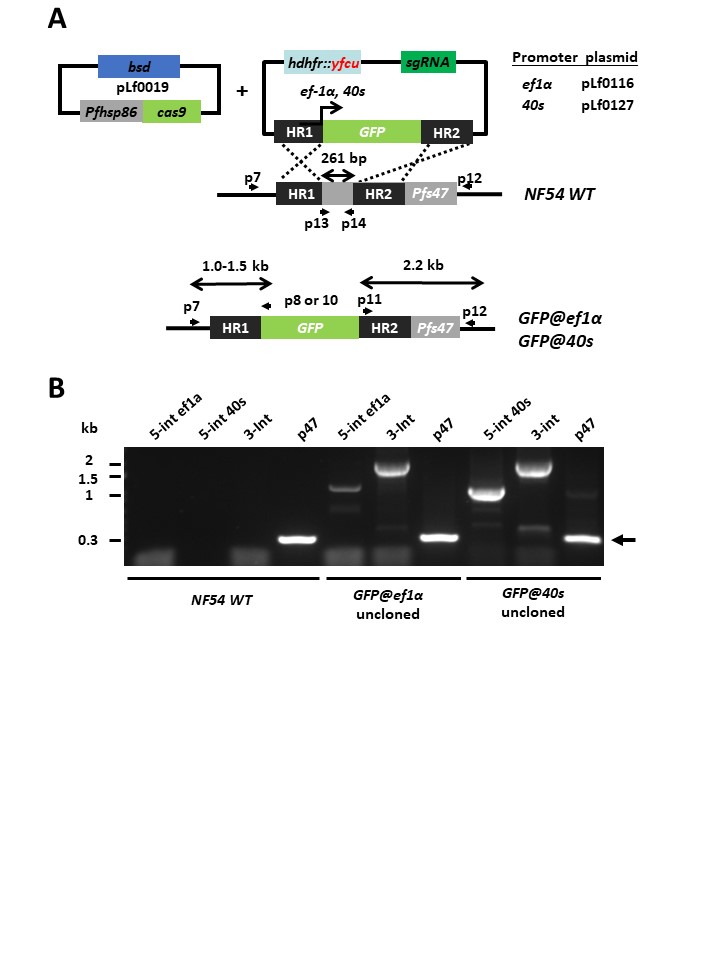

Supplement: Figure S2 — Generation of P. falciparum NF54 reporter lines expressing GFP under control of ef1α or 40s promoter. (A) Schematic representation of the Cas9 (pLf0019) and donor DNA plasmids (pL0116, pLf0127) constructs used to introduce the GFP expression cassette into the Pf NF54 p47 gene locus. The GFP gene is under the control of the promoter of the ef1α or 40s gene. The p47 homology regions (HR1, HR2) used to introduce the donor DNA (i.e., the GFP expression cassette), location of primers (p), and PCR amplicons (in black) are indicated. Primer sequences (shown in black and bold) are shown in Table S1. WT, wild type; bsd, blasticidin selectable marker hdhfr::yfcu—SM in donor plasmid. (B) Diagnostic PCR confirms the correct 5′ integration of the plasmids into the genome of GFP@ef1α and mCh-Luc@40s parasites (5-Int; primers p7/p8 for ef1α 1,009 bp, p7/p10 for 40s 1,087 bp) and correct 3′ integration (3-Int; primers p11/p12; 2,188 bp). Primer locations and product sizes are shown in (A) and primer sequences in Table S1. The arrow indicates PCR product of WT p47 gene amplified by p13/p14 primers (216 bp). [file Image_2.JPEG]

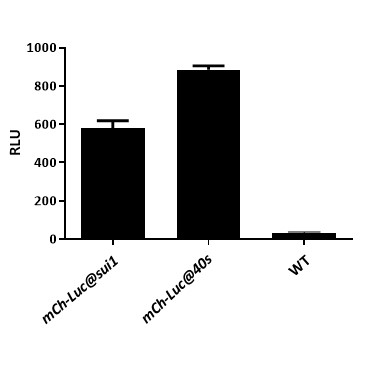

Supplement: Figure S3 — Luciferase expression in blood stages of mCh-Luc@sui1 and mCh-Luc@40s. Luciferase activity (RLU, relative light units) in asexual blood stages of mCh-Luc@sui1 (cl.3), mCh-Luc@40s (FACS), and wild type Pf NF54 parasites (WT). The mean luminescence value of triplicate samples is shown (1.0 × 10^6^ blood stages per sample); error bars represent the standard deviation. [file Image_3.JPEG]

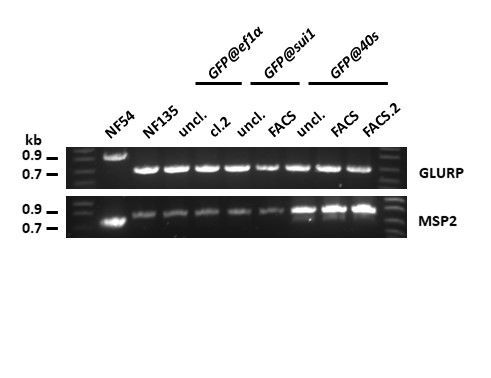

Supplement: Figure S4 — Diagnostic PCR on genomic DNA of GFP@ef1αNF135, GFP@sui1NF135, and GFP@40sNF135 to distinguish Pf NF54 and Pf NF135. Diagnostic PCR confirms the Pf NF135 genetic background of the GFP@ef1αNF135, GFP@sui1NF135, and GFP@40sNF135 lines. PCR amplification of the glurp gene (primers p15/p16) and the msp2 gene (primers p17/18) resulting in the expected PCR fragments of ~700 and ~900 bp, respectively in NF135 strain. [file Image_4.JPEG]

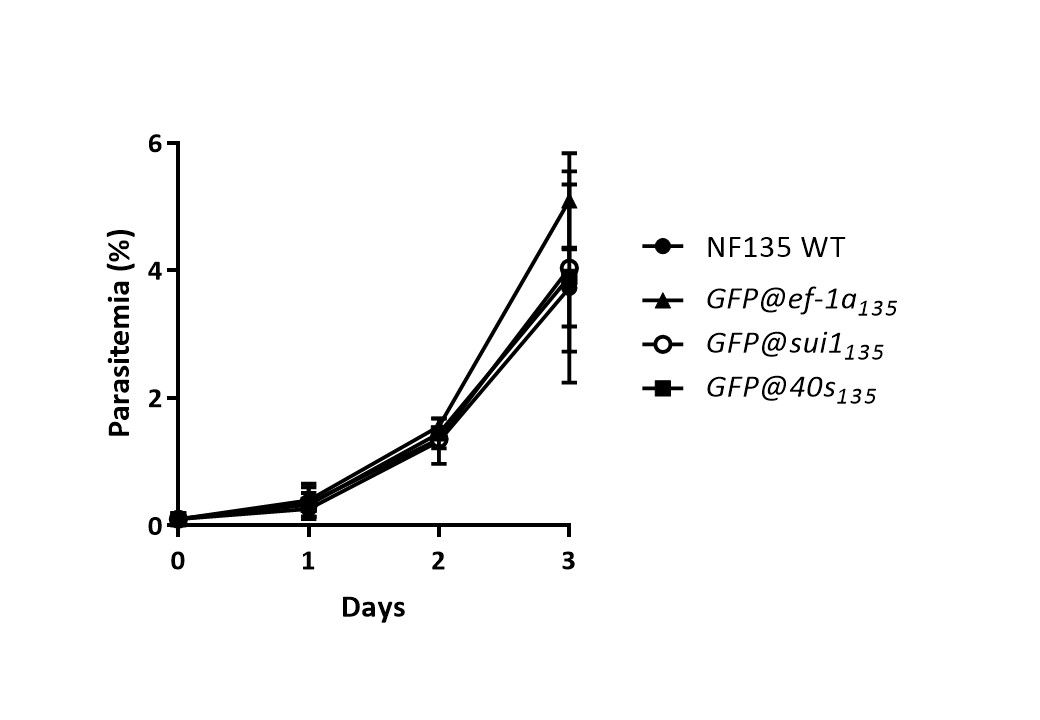

Supplement: Figure S5 — Characterization of the growth kinetics of asexual blood stages of GFP@ef1αNF135, GFP@sui1 NF135, and GFP@40sNF135. Growth of asexual blood-stages of GFP@ef1α clone2, GFP@sui1 (FACS sorted), and GFP@40s (FACS2 sorted) and wild type (WT) Pf NF135. Parasitemia (mean and S.D of three independent cultures) is shown during a 3-day culture period (in the semi-automated culture system). Cultures were initiated with a parasitemia of 0.1%. [file Image_5.JPEG]

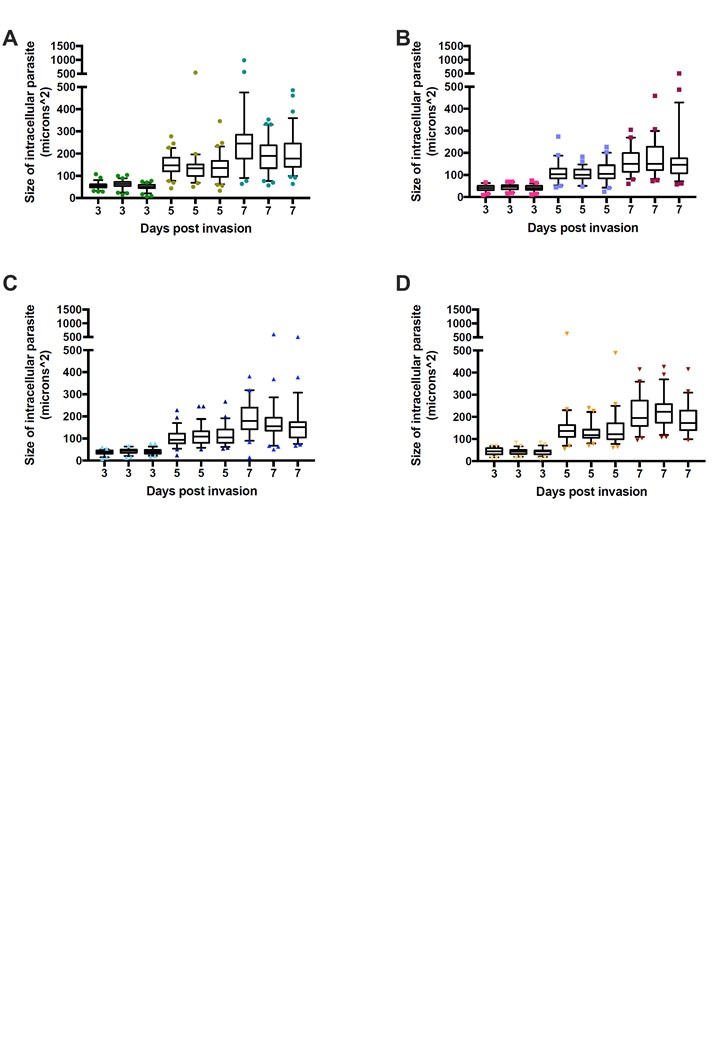

Supplement: Figure S6 — The size of three NF135 reporter lines in the fresh primary human hepatocytes (A). Sizes of Pf NF135 (A), GFP@40sNF135 (B), GFP@sui1NF135(C) and GFP@ef1αNF135 (D) intracellular parasites for three different wells at each time point. Each box plot is made up of measurements of at least 50 intracellular parasites. Horizontal line within each box plot represents the median and the dots shows value outside the 5–95% confidence interval. [file Image_6.JPEG]
